# Elisha Parsons

**Published:** 1889-11

**Authors:** E. M. Allen

**Affiliations:** Marietta, Ga.


### Obituary.

---

"Elisha Parsons, M. D., D. D. S., died at his residence, 115 York street, Savannah, Ga., on the 21st of August, 1889, in his 83d year.

"He was born in Northampton, Mass., November 29, 1806, which place he left in 1829 and went to Ohio. He graduated in medicine and surgery in Cincinnati, but not liking them, took the branch of dentistry as his profession, and to which he most ardently devoted himself.

"He settled in Savannah early in 1842, and was for years the leading pioneer dentist of his section. He ever strove to advance and elevate the tone of his profession, and was actively instrumental in forming the State Association. His growing feebleness incapacitated him for operative dentistry the last year of his life. In 1850 the Baltimore College of Dental Surgery conferred an honorary diploma on him for valuable services rendered to the profession of dentistry."

The above was furnished by a relative of the deceased, and conveys an insight into the early life of one whom the dentists delighted to honor, and I think they should give expression to the estimation in which he was held by the profession.

By request and as a representative of the Georgia State Dental Society, I have prepared the following testimonial of our regard for the oldest dentist in Georgia:

I first saw Dr. Parsons in Savannah in 1846, when he was patronized and beloved by the best families, and I have never known one who retained the practice in families to the second and third generations to a great extent. No man was more faithful and conscientious in the performance of his duties. While he was a dentist of the old school, he was not wedded to everything old, but was always willing to examine new materials and new methods of practice, and adopt whatever he could conscientiously endorse, his motto being "prove all things, and hold fast to that which is good."

When examining the teeth of persons I have met from Savannah, I have often inquired who inserted those good gold fillings, and the reply has frequently been, "Dr. Parsons, more than thirty years ago, and he is now operating for my grand children." Few in our profession have such a record.

After a successful practice he retired to a farm in Middle Georgia, previous to the late war. Being on the line of Sherman's march in 1864, his prospects in that direction were completely destroyed, and he returned to his practice in Savannah, to the delight of his friends.

At the close of the meeting, when the "Southern" was organized in Atlanta, in July, 1869, at an informal gathering of a few Georgia dentists, Dr. Burr was called to the chair, and it was decided to form a State society, and this meeting was adjourned to meet in Savannah in December following, at which time the Society was organized with twelve members, of whom Dr. Parsons was the oldest, and he always evidenced a lively interest in its welfare; one to whom all looked for example and advice. He was not absent from the annual meeting but twice previous to the last, when he was ill from which he did not recover. In 1886 he was also very sick, and the Society passed a resolution regretting the absence of such an earnest and faithful member. He was twice elected president which office he honored.

It has been remarked of more than one man that "he *talked* much but *said* nothing." When our deceased friend *talked* in our meetings, he was sure to say something; and his essays and reports expressed thoughts worthy of remembrance.

Besides furnishing articles for dental periodicals, he published several pamphlets for circulation among his patients, which contained instructive advice. His receipts, and results of experiments in laboratory, deserve a place in every dental office. No one has contributed more by his active influence and advice to elevate and sustain the professional character of dentists, and to promote among them mutual improve, social intercourse and good will.

His last letter to me was written in the Summer of 1888

just after the death of his estimable wife with whom he lived more than forty years, and when she died he said, "She left her love for every one who was lovable," and such, I doubt not, was his legacy also, for he possessed a warm heart and kindly feelings for all who could appreciate them.

The older members feel that they have lost a devoted brother, and the younger ones, a faithful friend.

A brother, Dr. E. E. Parsons, one son and a daughter and step-son (Col. Avery) remain with whom we deeply sympathize.

E. M. ALLEN.

Marietta, Ga., Sept. 24, 1889.—*Southern Dental Journal.*

